# Hsp21 Potentiates Antifungal Drug Tolerance in *Candida albicans*

**DOI:** 10.1371/journal.pone.0060417

**Published:** 2013-03-22

**Authors:** François L. Mayer, Duncan Wilson, Bernhard Hube

**Affiliations:** 1 Department of Microbial Pathogenicity Mechanisms, Hans-Knoell-Institute, Jena, Germany; 2 Center for Sepsis Control and Care, Universitätsklinikum, Jena, Germany; 3 Friedrich Schiller University, Jena, Germany; Institute of Microbiology, Switzerland

## Abstract

Systemic infections of humans with the fungal pathogen *Candida albicans* are associated with a high mortality rate. Currently, efficient treatment of these infections is hampered by the relatively low number of available antifungal drugs. We recently identified the small heat shock protein Hsp21 in *C. albicans* and demonstrated its fundamental role for environmental stress adaptation and fungal virulence. Hsp21 was found in several pathogenic *Candida* species but not in humans. This prompted us to investigate the effects of a broad range of different antifungal drugs on an Hsp21-null *C. albicans* mutant strain. Our results indicate that combinatorial therapy targeting Hsp21, together with specific antifungal drug targets, has strong synergistic potential. In addition, we demonstrate that Hsp21 is required for tolerance to ethanol-induced stress and induction of filamentation in response to pharmacological inhibition of Hsp90. These findings might pave the way for the development of new treatment strategies against *Candida* infections.

## Introduction

*Candida albicans* is one of the major fungal pathogens of humans and can cause life-threatening systemic infections with mortality rates approaching 50% [1]. Treatment of such infections is complicated due to the restricted number of efficient antifungal drugs, antifungal drug toxicity, and insufficient diagnostic tools [2], [3]. An emerging field of antifungal drug research is the combination of immunotherapeutic approaches with antifungal approaches, such as, for example, the combination of anti-Hsp90 antibodies with caspofungin, fluconazole, or amphotericin B [4], [5].

Three major classes of antifungals are used for the treatment of fungal infections: polyenes, which target ergosterol and cell membrane integrity; allylamines and azoles, which both block the ergosterol biosynthetic enzyme Erg11 (also known as lanosterol 14α-demethylase) and lead to an accumulation of toxic sterols; and echinocandins, which inhibit the β-1,3 glucan synthase and compromise cell wall integrity [6].

Heat shock proteins (Hsps) are found in virtually all living organisms, including humans and fungi. They fulfill a plethora of cellular functions, including folding, unfolding or refolding of other proteins (clients), translocation of client proteins across membranes, activation of clients, and prevention of uncontrolled protein aggregation [7]. Hsps are constitutively present within cells, however their expression rises dramatically upon stress; indeed, Hsp concentrations can reach over 20% of total cell protein [8]. Application of thermal stress to the model host *Drosophila melanogaster* led to the discovery of the heat shock response [9]. Later studies revealed that expression of specialized proteins, the Hsps, is strongly induced in response to heat and other forms of stress. Hsps are divided into five classes - Hsp100, Hsp90, Hsp70, Hsp60 and the small heat shock proteins (sHsps) - depending on their molecular mass [10], [11].

One of the most conserved and best investigated Hsps is Hsp90. Except for archaea, all living organisms encode at least one Hsp90 protein [12]. This essential chaperone is present in large quantities in cells even under non-stress conditions. Upon environmental stress, Hsp90 levels approximately double [12]. Hsp90 is a dimer and its function was shown to be ATP-dependent. Transcription of the *HSP90* gene is regulated by the transcription factor heat shock factor 1 (HSF1).

In humans, the function of Hsp90 has been associated with cancer. The chaperone was shown to protect cancer cells from extracellular stresses thereby promoting oncogenesis [7]. Consequently, Hsp90 has emerged as an attractive target for cancer treatment. Three different families of Hsp90 chaperones are found in humans. The first family, Hsp90 A, is localized to the cytoplasm and consists of Hsp90 AA1, Hsp90 AA2 and Hsp90 AB1. Hsp90 B forms the second class and contains the endoplasmic reticulum-localized chaperone, endoplasmin (also known as GRP-94). Finally, TRAP1 (also known as Hsp75) is present in mitochondria and is part of the TRAP family of Hsp90 proteins [7].

In *C. albicans*, the heat shock inducible *HSP90* gene was first investigated by the Brown laboratory and shown to be essential for viability [13]. Later work by the Cowen laboratory established that Hsp90 enables the emergence of drug resistance by stabilizing the protein phosphatase calcineurin and the mitogen activated protein (MAP) kinase Mkc1 [14]–[16]. Moreover, Hsp90 was shown to regulate biofilm dispersion and drug resistance and to be required for virulence [17], [18]. Because of the central role of Hsp90 in the *C. albicans* chaperone network, targeting Hsp90 has been proposed as an effective therapeutic strategy [19]. However, mammalian and fungal Hsp90 share a high degree of similarity and it was shown that targeting Hsp90 in mice results in serious toxic side-effects [19]. Human and murine Hsp90 share approximately 99% identity on the protein level. Hence, albeit not investigated, it is very likely that targeting *C. albicans* Hsp90 in human patients would also result in serious side-effects.

*C. albicans* occurs in three main morphological forms: yeast, pseudohyphal and hyphal cells. The transition from yeast to filamentous cells has been recognized as an important virulence trait in *C. albicans* as mutants defective in this transition are attenuated in virulence [20]. Several cues promote the yeast-to-hyphal switch including serum, temperatures of 37°C or higher, a high pH (> 7) and low cell densities (< 10^7^ cells ml^-1^) [21]. The Cowen laboratory elegantly demonstrated that compromising Hsp90 function, either genetically or pharmacologically, results in filament formation under non-hypha-inducing conditions [18]. Indeed, the authors found Hsp90 to repress one of the key hyphae-inducing pathways, the cAMP-PKA signaling pathway, under these conditions.

In comparison to Hsp90 and the other higher molecular mass Hsps, the class of small Hsps has historically received only little attention. The only two sHsps that have recently been investigated in *C. albicans* are Hsp12 and Hsp21. Despite being strongly upregulated in response to a wide variety of environmental stresses, both on a transcriptional and protein level, Hsp12 was shown to be dispensable for stress resistance, morphogenesis and virulence in a *Drosophila* model of infection [22]. Hsp21 is also strongly induced upon various environmental stresses [23]–[29]. We demonstrated that Hsp21 is required for thermal and oxidative stress tolerance in *C. albicans* [30]. Moreover, Hsp21 was required for normal filamentation, regulation of intracellular levels of the stress-protective molecule trehalose, and activation of the mitogen-activated protein (MAP) kinase Cek1. An *hsp21*Δ/Δ mutant had impaired capacity to damage endothelial and oral epithelial cells *in vitro*, had increased sensitivity to human neutrophils, and was strongly attenuated in virulence in two *in vivo* infection models: an embryonated hen egg infection model and a mouse infection model of hematogenously disseminated candidiasis [30].

Here, we have investigated the suitability of Hsp21 as a novel therapeutic target for the treatment of candidiasis. We demonstrate strong synergistic effects between Hsp21 inactivation and specific antifungal drug treatment. Moreover, we show that whilst *HSP21* orthologues are present in the majority of pathogenic *Candida* species, the gene is not found in humans. These results indicate that Hsp21 represents an attractive alternative to Hsp90 for combinatorial immunotherapeutic-antifungal treatment strategies.

## Results

### Phylogenetic relatedness of *C. albicans* Hsp21 and human sHsps

**Figure 1:**
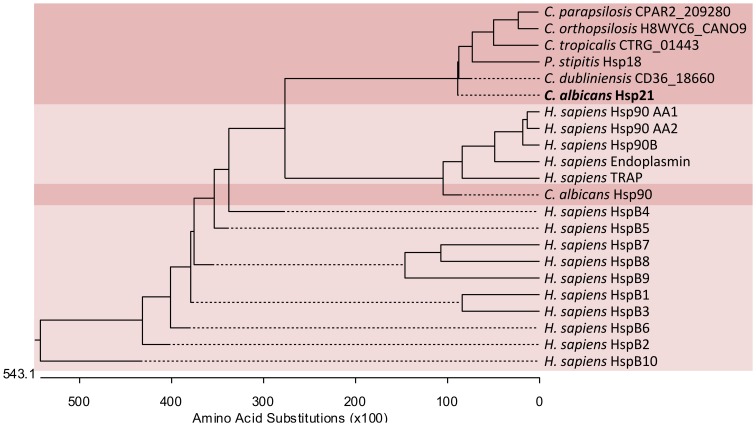
Phylogenetic relationship of *C. albicans* Hsp21 and Hsp90 with human sHsps and Hsp90 proteins. *C. albicans* Hsp90 is closely related to the five human Hsp90 proteins. Hsp21 from *C. albicans* is only distantly related to the 10 known sHsps of humans, but is similar to uncharacterized proteins from *C. dubliniensis*, *C. tropicalis*, *C. parapsilosis*, *C. orthopsilosis*, and to the uncharacterized sHsps Hsp18 from *P. stipitis*. HspB1-10, human heat shock protein beta 1–10; Hsp90 AA1/2, heat shock protein 90 alpha class A member 1/2; Hsp90 AB1, heat shock protein 90 alpha class B member 1; TRAP1, tumor necrosis factor type 1 receptor-associated protein.

Targeting the molecular chaperone Hsp90 in *C. albicans* has been proposed to be an attractive strategy to combat infection. Indeed, compromising Hsp90 leads to reduced biofilm formation [17], increased sensitivity to antifungal drugs [14], [16], and attenuated virulence of *C. albicans* in a mouse model of hematogenously disseminated candidasis [14], [18], [19]. However, a considerable drawback to targeting this fungal Hsp in a clinical setting is the cross-reactivity with human Hsps. Humans encode five Hsp90 proteins: Hsp90 AA1, Hsp90 AA2, Hsp90 AB1, endoplasmin, and TRAP1. CaHsp90 shares 60–70% identity with human Hsp90 AA1, Hsp90 AA2 and Hsp90 AB1, around 47% identity with endoplasmin and approximately 34% identity with TRAP1 on the protein level (Figure 1 and Table 1). This significant overlap in sequence prompted us to search for alternative / additional fungal Hsp targets. Recently, we have identified and characterized the sHsp Hsp21 in *C. albicans* [30]. Deletion of *HSP21* negatively affected environmental stress tolerance, resulted in reduced capacity to damage endothelial and oral epithelial cells *in vitro*, and strongly attenuated virulence in a mouse model of hematogenously disseminated candidasis [30]. Humans encode 10 sHsps, HspB1-10 [31]. Alignments revealed that Hsp21 is only distantly related to the 10 human sHsps (Figure 1) and the percentage identities on the protein level were only 11–15% (Table 1). Moreover, BLASTp analysis of the Hsp21 protein sequence directly against the human proteome identified the anion exchange transporter, SUT2, as best hit. An alignment of the Hsp21 and SUT2 protein sequences revealed 12.7% identity between both proteins. In contrast, *C. albicans* Hsp21 shares 96%, 53%, 40%, and 38% identity with uncharacterized proteins from *Candida dubliniensis*, *Candida tropicalis*, *Candida parapsilosis*, and *Candida orthopsilosis*, respectively, and 39% identity with the uncharacterized sHsp Hsp18 from *Pichia stipitis* (Figure 1 and [30]). These results indicate that Hsp21 might represent an attractive anticandidal target as it is present in several pathogenic *Candida* species, but not found in humans.

**Table 1:** Similarity of *C. albicans* Hsp21 and Hsp90 with human sHsps and Hsp90 proteins.

C. albicans	Homo sapiens	Identity (%)
Hsp90	Hsp90 AA1	63.5
	Hsp90 AA2	69.0
	Hsp90 AB1	70.3
	Endoplasmin	47.5
	TRAP1	34.3
Hsp21	HspB1	12.0
	HspB2	15.3
	HspB3	11.1
	HspB4	15.4
	HspB5	14.7
	HspB6	14.1
	HspB7	14.9
	HspB8	11.4
	HspB9	10.9
	HspB10	12.1

The *C. albicans* Hsp90 and Hsp21 protein sequences were retrieved from the *Candida* Genome Database (CGD). Human sHsp and Hsp90 protein sequences were retrieved from the UniProt database. Sequences were aligned and analysed for percentage identity using the Clustal W method in the DNASTAR Lasergene MegAlign sequence analysis software.

### Hsp21 contributes to resistance against ethanol-induced stress

**Figure 2:**
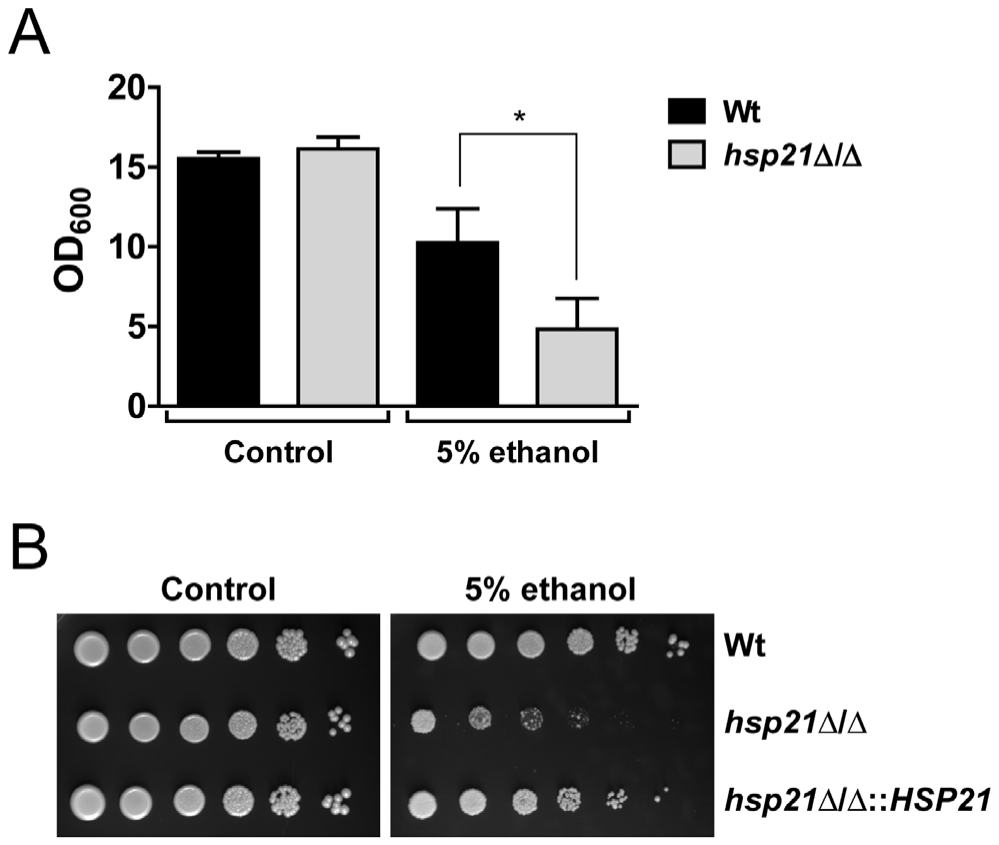
Hsp21 contributes to cell membrane directed stress tolerance. The *hsp21*Δ/Δ mutant is more susceptible to ethanol-induced cell membrane stress in comparison to the wild type (Wt). **(A)** Fungal YPD-overnight cultures were adjusted to OD_600_  =  1 in YPD alone or YPD supplemented with 5% ethanol. Strains were incubated for 24 h at 30°C and 210 rpm in a shaking incubator and the OD_600_ was determined. Results are the mean ± SD of three independent experiments. *P<0.05. **(B)** Serial drop dilution assays on SD agar or SD agar supplemented with 5% ethanol. Plates were incubated at 37°C for 2–6 days. The experiment was repeated twice in duplicate. Representative pictures are shown.

Most antifungal agents in clinical use target cell membrane localized ergosterol, ergosterol biosynthesis or biosynthesis of β-1,3 glucan, which is a major constituent of the fungal cell wall [32]. Ergosterol is a fungal specific sterol present in cell membranes and required for cell membrane permeability and fluidity. We previously found that an *hsp21*Δ/Δ mutant had normal resistance to cell wall directed stresses [30]. We therefore investigated the effects of ethanol (which induces cell membrane disturbances and protein unfolding) on growth of the mutant. Following growth in liquid YPD medium supplemented with 5% ethanol, the *hsp21*Δ/Δ mutant (final OD_600_  =  4.8) was found to be significantly more susceptible to ethanol-induced stress in comparison to the wild type (final OD_600_  =  10.2) (Figure 2A). Additionally, the *hsp21*Δ/Δ mutant displayed a strong growth defect under ethanol stress on agar-containing medium in comparison to the wild type and revertant (*hsp21*Δ/Δ::*HSP21*) (Figure 2B). These results indicate that Hsp21 contributes to ethanol stress tolerance in *C. albicans*, and suggest that Hsp21 might also be required for normal resistance to antifungal drugs which target the fungal cell membrane.

### Hsp21 potentiates resistance to antifungal drugs

**Figure 3:**
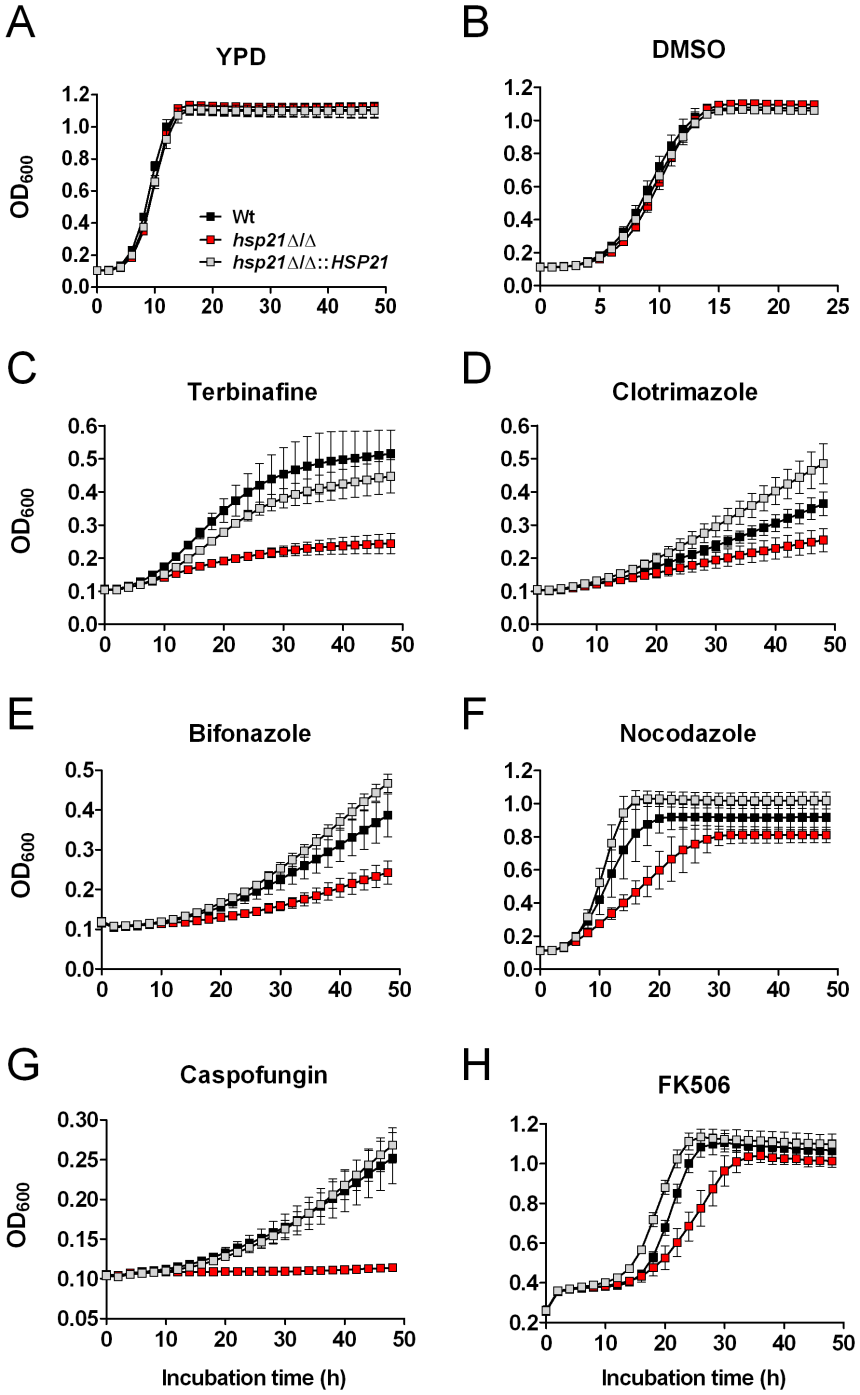
Hsp21 potentiates antifungal drug resistance in *C. albicans*. Growth curves of the indicated strains in YPD medium **(A)**, YPD medium supplemented with dimethyl sulfoxide (DMSO) **(B)**, YPD medium supplemented with 10 µg ml^-1^ terbinafine **(C)**, 1 µM clotrimazole **(D)**, 1 µM bifonazole **(E)**, 5 µg ml^-1^ nocodazole **(F)**, 2 µg ml^-1^ caspofungin **(G)**, or YPD medium supplemented with 250 µg ml^-1^ FK506 **(H)**. Growth was recorded in an ELISA reader at 37°C for the indicated time. Results are the mean ± SD of two independent experiments, each performed in quadruplicate.

**Figure 4:**
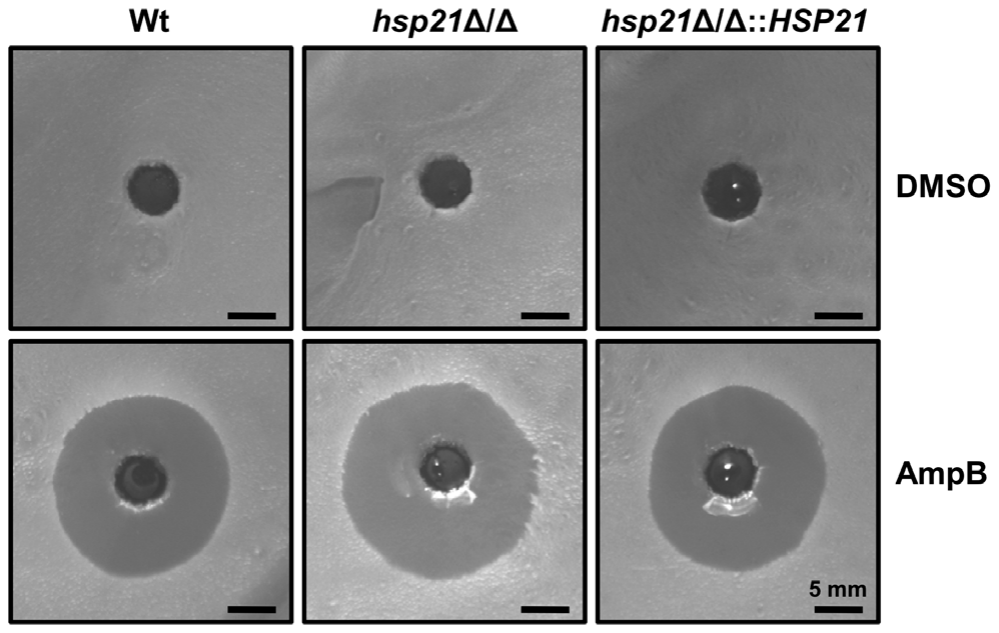
Compromising Hsp21 results in moderately increased susceptibility towards amphotericin B. Amphotericin B susceptibility was assessed with a drug diffusion assay. 4×10^7^ cells of the indicated strains were plated on SD agar and two holes of approximately 5 mm in diameter were generated and filled with 10 µl DMSO, or 10 µl amphotericin B (AmpB, 1 mg ml^-1^), respectively. The plates were incubated at 37°C for 24 h and then photographed. The zone of growth inhibition around AmpB-treated holes is larger for the *hsp21*Δ/Δ mutant in comparison to the wild type (Wt) and reconstituted strain.

We next explored the effects of a comprehensive range of antifungal drugs on growth of the *hsp21*Δ/Δ mutant (Figures 3 and 4). Drugs targeting biosynthesis of ergosterol included the allylamine, terbinafine, and the imidazoles, clotrimazole and bifonazole. In order to directly target ergosterol we used amphotericin B. Furthermore, we included antifungal drugs directed against β-1,3 glucan biosynthesis (caspofungin), or microtubuli (nocodazole).

In YPD medium (Figure 3A) or YPD medium supplemented with the drug vehicle DMSO (Figure 3B) the wild type, *hsp21*Δ/Δ mutant, and *hsp21*Δ/Δ::*HSP21* complemented strain displayed very similar growth. In YPD medium supplemented with 10 µg ml^-1^ terbinafine, the *hsp21*Δ/Δ mutant exhibited strongly reduced growth in comparison to the wild type and complemented strain (Figure 3C). In the presence of 1 µM clotrimazole (Figure 3D) or bifonazole (Figure 3E), the *hsp21*Δ/Δ mutant also exhibited reduced growth in comparison to the wild type and revertant. In the presence of 5 µg ml^-1^ nocodazole, which targets fungal microtubuli, the *hsp21*Δ/Δ mutant exhibited impaired growth in comparison to the wild type and complemented strain (Figure 3F). Exposure to 2 µg ml^-1^ caspofungin resulted in complete growth inhibition of the *hsp21*Δ/Δ mutant (Figure 3G). In contrast, both the wild type and revertant strain were able to grow under these conditions. To directly target ergosterol, the strains were exposed to amphotericin B in a drug diffusion assay (Figure 4). While exposure to the vehicle DMSO did not inhibit growth of either strain, application of 1 mg ml^-1^ amphotericin B resulted in growth inhibition zones for all three strains (Figure 4). While the inhibition zones were comparable between the wild type and revertant strain (approximately 18 mm in diameter), the zone of inhibition was slightly larger for the *hsp21*Δ/Δ mutant (approximately 20 mm in diameter).

These results indicate that Hsp21 potentiates tolerance to commonly used antifungal drugs in *C. albicans*.

### Targeting Hsp21 renders *C. albicans* partially susceptible to FK506

Transcriptional data suggested that *HSP21* lies downstream of the cyclic AMP (cAMP) pathway [33]. The major molecular chaperone Hsp90 regulates the GTPase Ras1, which in turn regulates the cAMP pathway member, Cyr1 (adenylate cyclase). Hence, Hsp90 directly affects the cAMP pathway. An important client protein chaperoned by Hsp90 is the protein phosphatase calcineurin [14], [16]. It has been demonstrated that calcineurin is essential for surviving cell membrane stress [34]. For example, the combination of the calcineurin inhibitor, FK506, with the ergosterol biosynthesis-inhibiting azole, fluconazole, resulted in potent synergistic antifungal activity [34], [35].

We therefore investigated whether simultaneously targeting Hsp21 (which contributes to cell membrane integrity, see above) and inhibiting calcineurin with the drug FK506 might result in a similar synergistic effect. Indeed, the *hsp21*Δ/Δ mutant exhibited markedly delayed growth in the presence of FK506 (Figure 3H). These results indicate that deletion of *HSP21* renders *C. albicans* partially susceptible to FK506.

### Hsp21 contributes to Hsp90-inhibition induced filamentation

It has recently been shown that Hsp90 acts as physiological link between *C. albicans* morphogenesis and temperature [18], [36], [37]. Three findings prompted us to investigate Hsp90-inhibition induced filamentation in the *hsp21*Δ/Δ mutant: first, Hsp21 contributes to normal filamentation in *C. albicans* [30]; second, Hsp21 was required for optimal growth upon inhibition of the Hsp90-regulated phosphatase, calcineurin (see above); and, third, transcriptional data indicates that Hsp21 and Hsp90 are both part of the cAMP pathway [33].

**Figure 5:**
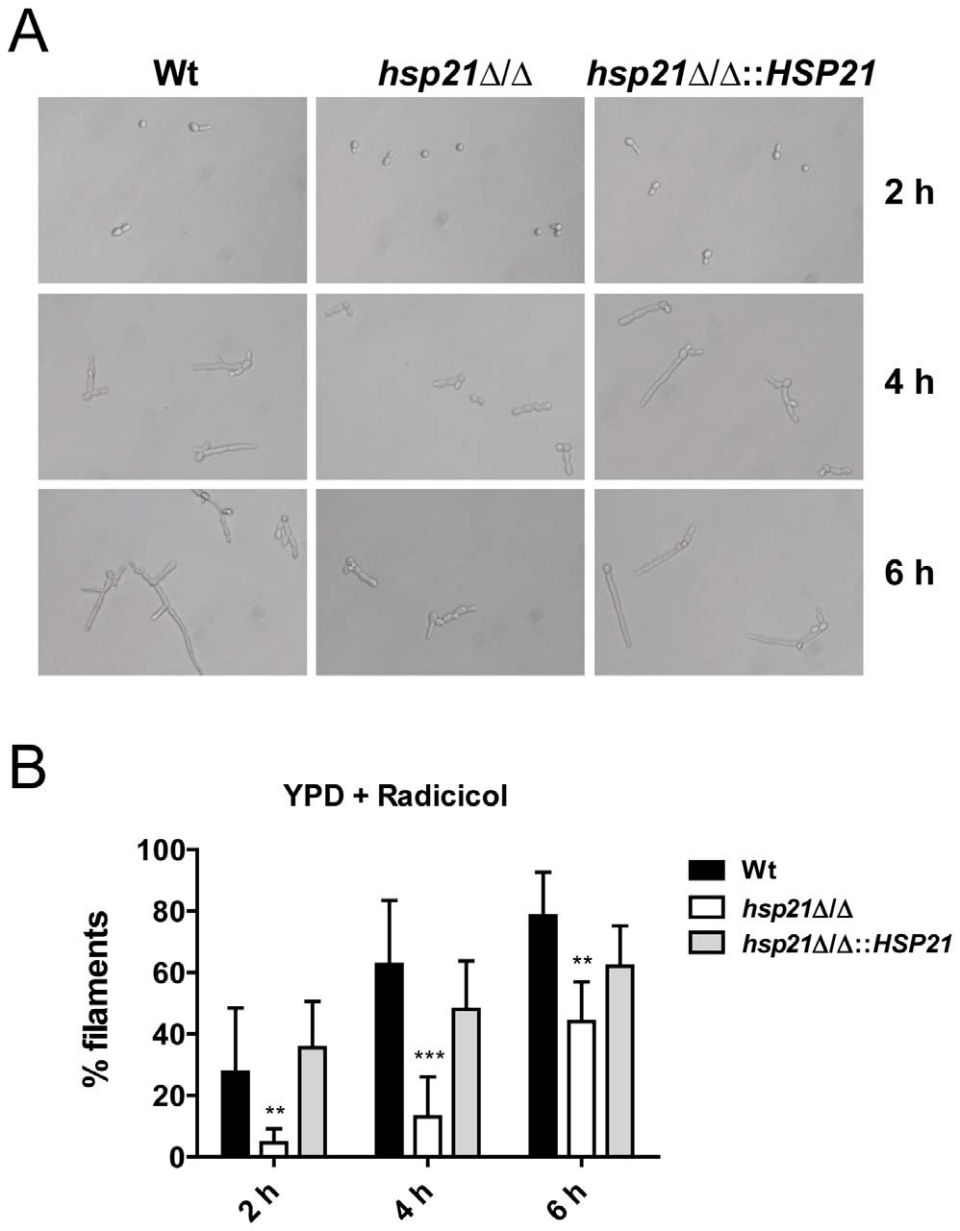
Hsp21 contributes to Hsp90 inhibition-induced filamentation. Analysis of Hsp90 inhibition-induced filamentation dynamics in the *hsp21*Δ/Δ mutant. **(A)** YPD overnight cultures of the wild type (Wt), *hsp21*Δ/Δ mutant and *hsp21*Δ/Δ::*HSP21* complemented strain were subcultured in fresh YPD medium supplemented with 27 µM radicicol and incubated for the indicated time at 37°C. Representative pictures were captured with an inverse microscope (Leica DMIL). **(B)** Quantification of the percentage filamentation from (A). Results are the mean ± SD of two independent experiments, each performed in quadruplicate. At least 50 random cells per strain and experiment were examined. **P<0.01 and ***P<0.001 compared with the wild type and *hsp21*Δ/Δ::*HSP21* complemented strain.

Therefore, the effect of the Hsp90 inhibitor radicicol on filamentation of *hsp21*?/? was investigated. The *hsp21*Δ/Δ mutant displayed strongly delayed germ tube formation in comparison to the wild type and revertant (Figure 5A and B). While approximately 30% of wild type and revertant cells formed germ tubes after 2 h exposure to radicicol, only around 5% of *hsp21*Δ/Δ cells had begun to filament (Figure 5A and B). After 4 h incubation, 63% of wild type and 48% of revertant cells had formed germ tubes and started to form longer filaments. In contrast, only around 13% of *hsp21*Δ/Δ cells had formed short germ tubes and longer filaments were not yet present (Figure 5A and B). Following 6 h incubation, 78% of wild type and 62% of revertant cells had filamented. At this time point, although 44% of *hsp21*?/? cells had filamented (Figure 5B), these were markedly shorter than wild type and revertant cells (Figure 5A).

Taken together, these results indicate that Hsp21 plays a role in filament induction in response to Hsp90-inhibition.

## Discussion

In the current study, we have evaluated the suitability of targeting fungal heat shock proteins as a potential therapeutic strategy. Fungal Hsp90 has previously been investigated as a immunotherapeutic target; however, cross-reactivity against mammalian Hsp90 proteins has hampered the development of anti-Hsp90 therapy for the treatment of fungal disease [19].

A recent quantitative analysis revealed that the major molecular chaperone Hsp90 interacts with nearly 400 client proteins in humans (almost 2% of the proteome) [38]. Hsp90 regulates the shape and function of many important signal transducers, mainly kinases, E3 ligases, and transcription factors [38]. Together with other Hsps, Hsp90 prevents unfolding and aggregation of client proteins both under non-stress conditions and upon environmental stresses, thereby ensuring cell survival.

Hsps are strongly expressed in human cancer cells and protect these cells from environmental insults [7]. Therefore, in the setting of cancer, the chaperoning functions of Hsps, and in particular Hsp90, actually promote tumour cell survival and proliferation. As a consequence, targeting human Hsp90 in human tumours has been proposed to be an attractive anti-cancer strategy [7].

In *C. albicans*, Hsp90 regulates antifungal drug resistance, biofilm formation and virulence, and interacts with at least 200 distinct proteins [16]–[18], [39]. In addition, Hsp90 regulates temperature-dependent filamentation and thereby directly contributes to the virulence potential of the fungus [18]. Hence, targeting Hsp90 in *C. albicans* has been proposed to be a promising antifungal strategy. However, Hsp90 from *C. albicans* is very similar to human Hsp90. Therapeutic targeting of fungal Hsp90 would therefore result in significant side-effects for patients due to simultaneous inhibition of human Hsp90. Moreover, humans encode five Hsp90 proteins which all display significant similarity to the fungal counterpart (Table 1 and Figure 1). Therefore, the side-effects of targeting *C. albicans* Hsp90 would probably not just be restricted to one human protein, but would extend to a whole protein family and their respective cellular circuitries. Mice contain four Hsp90 proteins and experiments in a mouse model of disseminated candidiasis with Hsp90 inhibitors indicated significant toxicity for the murine host, thereby precluding application of such an inhibitor in humans [19]. Efforts are therefore centered on two refined approaches in Hsp90-targeting. First, fungal-selective inhibitors might be developed which specifically target Hsp90 protein sites that are divergent between human and fungi, e.g. the N-terminal ATPase site [19]. Second, targeting fungal specific regulators of Hsp90 function, such as lysine deacetylases, have recently been proposed to be an attractive alternative to direct Hsp90 targeting [40].

In this work we propose that targeting fungal-specific Hsps might represent an additional / alternative strategy to combat *Candida* infections. We propose that the *C. albicans* sHsp Hsp21 is a promising candidate for such an approach for several reasons. First, we have previously demonstrated that deletion of *HSP21* strongly impairs the capacity of *C. albicans* to damage both endothelial and oral epithelial cells *in vitro*, reduces tolerance to the killing activities of neutrophils, and drastically reduces virulence in an *in vivo* mouse model of hematogenously disseminated candidiasis [30]. Second, in this study we establish that targeting Hsp21 results in significantly enhanced efficacy of most currently used antifungal drugs (Figure 3 and 4). Third, Hsp21 is not found in humans and displays only distant relation to human sHsps. And fourth, Hsp21 orthologues are found in the clinically relevant fungal pathogens *C. dubliniensis*, *C. tropicalis*, and *C. parapsilosis* [30]. Potential anti-Hsp21 drugs therefore would fulfill the most important requirements of a novel anticandidal compound, including specificity against pathogenic fungal cells only, few expected side-effects for patients, and efficacy against several pathogenic fungi. It should be noted, however, that Hsp21 is not found in other important fungal pathogens of humans, including *Candida glabrata*, *Aspergillus fumigatus*, *Cryptococcus neoformans*, or *Coccidioides immitis*. Nevertheless, combined with accurate diagnostics, or rational prophylaxis, targeting such a fungal virulence factor may represent an effective treatment strategy.

Deletion of Hsp90 in *C. albicans* has been demonstrated to abrogate the emergence of drug resistance [41]. It will be intriguing to investigate a potential role of Hsp21 in the evolution of antifungal drug resistance.

Small heat shock proteins have been proposed to prevent deleterious protein aggregation of partially unfolded proteins under environmental stress conditions by binding these clients in a sponge-like manner. In cooperation with major Hsps, such as Hsp70 or Hsp104, these clients are then either refolded or passed on to the degradation machinery for removal. Although the exact mechanistic function of Hsp21 remains to be elucidated, evidence suggests that this sHsp regulates intracellular levels of trehalose, possibly by activation of the mitogen-activated protein kinase Cek1 [30].

In the present investigation, we establish that Hsp21 potentiates the resistance of *C. albicans* towards several antifungal drugs. Deletion of Hsp21 resulted in strongly reduced growth rates in the presence of terbinafine, an allylamine that targets biosynthesis of ergosterol (Figure 3C). Ergosterol is the fungal-specific counterpart of human cholesterol and promotes cell membrane rigidity [42]. These results fit well with the finding that an *hsp21*Δ/Δ mutant had strongly reduced tolerance towards ethanol-stress which perturbs the plasma membrane (Figure 2). It should be mentioned, however, that ethanol also induces protein unfolding, besides affecting membrane fluidity [43]. Hsp21 was previously shown to regulate intracellular levels of the stress-protective molecule trehalose under environmental stress conditions [30]. Interestingly, *Saccharomyces cerevisiae TPS1* mutants (which are unable to synthesize trehalose), are unable to grow in the presence of ethanol [44]. A role of Hsp21 in stabilizing client proteins during ethanol-induced stress, either directly or via regulation of trehalose, can therefore not be excluded at this stage.

The clinically used azoles, clotrimazole and bifonazole, also target the biosynthesis of ergosterol. In addition, both azoles were shown to specifically affect the capacity of *C. albicans* to damage vaginal epithelial cells, while not affecting adhesion or invasion rates [45]. Growth rates of the *hsp21*Δ/Δ mutant were reduced in the presence of both drugs (Figure 3D and 3E). Direct targeting of ergosterol with amphotericin B also led to moderately enhanced sensitivity of an Hsp21-deleted strain (Figure 4). These results support a role for Hsp21 in cell membrane integrity.

Nocodazole targets microtubuli and we found that deletion of *HSP21* delays growth of the fungus in the presence of this drug (Figure 3F), suggesting that Hsp21 contributes to the integrity of the microtubular system under conditions of stress.

Although deletion of Hsp21 did not affect resistance to cell wall disturbing agents such as Congo red or calcofluor white [30], we now show that Hsp21 is required for growth in the presence of caspofungin, an antifungal agent which targets integrity of the fungal cell wall (Figure 3). Caspofungin specifically targets the biosynthesis of the cell wall by inhibiting the β-1,3 glucan synthase, Fks1. Caspofungin belongs to the echinocandin class of antifungals and is amongst the most recent drugs to reach the clinic in decades. Importantly, an *hsp21*Δ/Δ mutant was unable to grow in the presence of caspofungin under the conditions tested (Figure 3G), suggesting that combinatorial therapy with an Hsp21-inhibitor and caspofungin may be particularly effective.

Filamentation has been defined as a key virulence attribute in *C. albicans* [46], and the Cowen laboratory has demonstrated that the major chaperone Hsp90 controls this process in a temperature-dependent manner [18]. We found that deletion of *HSP21* and simultaneous pharmacological inhibition of Hsp90 resulted in significantly reduced germ tube and filament formation at early time points (Figure 5). These results suggest that Hsp21 contributes to filamentation in response to Hsp90 inhibition in *C. albicans* and support previous findings that these two Hsps function in the same pathway [18], [33].

The Hsp90 client protein calcineurin is inhibited by the drug FK506 [47]. Combining anti-calcineurin treatment with antifungal drugs results in a synergistic effect and efficiently kills *C. albicans* [34], [35], [48], [49]. We found that simultaneous deletion of *HSP21* and treatment with FK506 resulted in a moderate synergistic effect on growth of the fungus (Figure 3H). This result strengthens the concept that Hsp21 functions in the same pathway as Hsp90. Interestingly, it has recently been shown that the Hsp90 client protein Sgt1 is also involved in azole and echinocandin resistance, providing a further link between Hsp90 signalling and tolerance towards antifungals [50].

In summary, we have established that Hsp21 represents a promising novel anti-*Candida* target which could be used as part of a combinatorial strategy together with certain conventional antifungal drug treatment.

## Materials and Methods

### Strains and growth conditions

The triple-auxotrophic strain BWP17 complemented with plasmid CIp30 [51] was used as wild type control in all experiments. The *hsp21*Δ/Δ mutant and *hsp21*Δ/Δ::*HSP21* complemented strain have been published previously [30]. Strains were routinely grown on YPD agar [1% yeast extract, 2% bacto-peptone, 2% D-glucose, 2% agar] or SD minimal medium agar [2% dextrose, 0.17% yeast nitrogen base, 0.5% ammonium sulfate, 2% agar]. Overnight liquid cultures were grown in YPD medium in a shaking incubator at 30°C and 180 rpm. For growth curves, overnight cultures were diluted to an OD_600_ of 0.1 in 200 µl final volume of the desired medium. Growth of the strains was then recorded in sealed 96-well plates by measuring the OD_600_ at 30 min intervals for up to 50 hours in an ELISA reader (Infinite M200, Tecan) [52]. Experiments were performed twice in quadruplicate.

### Ethanol stress

YPD-overnight cultures were adjusted to an OD_600_ of 1 in YPD alone or YPD supplemented with 5% ethanol. Strains were then incubated for 24 h at 30°C and 210 rpm in a shaking incubator, followed by determination of the OD_600_. The experiment was performed three times.

### Antifungal drug treatments

The effects of different antifungal drugs on the growth rate of *C. albicans* strains were investigated. Growth curves were recorded in an ELISA reader at 37°C. Antifungal drug concentrations were: 10 µg ml^-1^ terbinafine (Sigma-Aldrich), 1 µM clotrimazole (Bayer AG), 1 µM bifonazole (Bayer AG), 5 µg ml^-1^ nocodazole (Sigma-Aldrich), and 2 µg ml^-1^ caspofungin (Cancidas, Merck & Co., USA). For inhibition of the calcineurin phosphatase, 250 µg ml^-1^ FK506 (AppliChem) were added to liquid YPD medium. Experiments were performed twice in quadruplicate.

Amphotericin B susceptibility was assessed with a drug diffusion assay. YPD overnight cultures of the respective strains were adjusted to 10^8^ cells ml^-1^ and 400 µl of this suspension plated onto SD agar. Two holes of approximately 5 mm in diameter were made and filled with 10 µl DMSO, or 10 µl Amphotericin B (1 mg ml^-1^, Sigma-Aldrich), respectively. The plates were incubated at 37°C for 24 h and then photographed. The experiment was repeated twice in duplicate yielding similar results.

### Serial dilution drop tests

Aliquots of overnight YPD cultures were washed twice in phosphate buffered saline (PBS) and 10-fold serial dilutions in 5 µl (covering a range of 10^6^ to 10^1^ cells) were spotted onto SD agar, or SD agar containing 5% ethanol and incubated at 37°C for 2-6 days. The experiment was repeated twice in duplicate.

### Radicicol-induced filamentation

YPD overnight cultures were subcultured in fresh YPD medium or YPD medium supplemented with 27 µM radicicol (A.G. Scientific, San Diego, USA) in 24-well plates and incubated for 2, 4 or 6 hours at 37°C. Experiments were performed in quadruplicate and repeated twice. Pictures were taken with an inverse microscope (Leica DMIL) and at least 50 randomly chosen cells per strain and experiment were examined for filamentation.

### Phylogenetic analysis

The phylogenetic tree was constructed using the Clustal W method in the DNASTAR Lasergene MegAlign sequence analysis software. All protein sequences were retrieved from CGD’s (*Candida* Genome Database) Multi-Genome Search database and from the Universal Protein Resource Knowledgebase (UniProtKB).

### Statistics

The Student’s t-test was applied for statistical analysis using GraphPad Prism version 5.00. *P*-values < 0.05 were considered to be significant.
